# The Efficacy of Cryotherapy in Reducing Pain and Anxiety During Venipuncture: A Systematic Review and Meta‐Analysis of Randomized Controlled Trials

**DOI:** 10.1155/prm/9474988

**Published:** 2026-11-09

**Authors:** Malaika Shams, Arsalan Ahmed, Nabahat Shafi, Hafiz Mustafa Anjum, Jawaria Firdous, Nayeemul Islam, Hafiz Muhammad Moaaz Sajid, Misal Fatima, Muhammad Adil Abid, Zeinab Hamdi Rezk

**Affiliations:** ^1^ Department of Medicine Nowshera Medical College Khyber Medical University Nowshera Pakistan kmu.edu.pk; ^2^ Department of Medicine Bilawal Medical College Liaquat University of Medical and Health Sciences Jamshoro Pakistan lumhs.edu.pk; ^3^ Department of Medicine Dow Medical College Dow University of Health Sciences Karachi Pakistan duhs.edu.pk; ^4^ Department of Medicine Federal Medical College Shaheed Zulfiqar Ali Bhutto Medical University Islamabad Pakistan szabmu.edu.pk; ^5^ Department of Medicine Punjab Medical College Faisalabad Medical University Faisalabad Pakistan pmc.edu.pk; ^6^ Department of Medicine Sir Salimullah Medical College Dhaka Bangladesh duhs.edu.pk; ^7^ Department of Allied Health Sciences Northwest Institute of Health Sciences Khyber Medical University Peshawar Pakistan kmu.edu.pk; ^8^ Faculty of Medicine Port Said University Port Said Egypt psu.edu.eg

## Abstract

**Background:**

Cold therapy, or cryotherapy, is the application of low temperature to reduce pain and swelling, and it is used widely in routine practice to ease the pain and anxiety of venipuncture. This systematic review and meta‐analysis evaluated its effect on venipuncture pain and anxiety, together with postprocedure heart rate.

**Methods:**

This systematic review and meta‐analysis was registered on PROSPERO and included randomized controlled trials (RCTs) of cryotherapy during venipuncture, with no geographical restrictions. We searched PubMed, the Cochrane Library, Embase, Scopus, Web of Science, and ClinicalTrials.gov, which returned 502 records, and screened them in Rayyan. Sixteen RCTs met the eligibility criteria, and 14 provided data suitable for pooling. Pain was the primary outcome, and anxiety was the secondary outcome, with postprocedure heart rate as an additional outcome. Risk of bias was assessed with the Cochrane ROB2 tool. Analyses were carried out in R using the meta and metafor packages with a random‐effects model and the Hartung–Knapp adjustment. Because the trials used several pain scales, the standardized mean difference (Hedges g) was the primary effect measure. Subgroup analyses and random‐effects meta‐regression (age, application duration, and needle gauge) were used to explore heterogeneity, and small‐study effects were examined with the funnel plot, Egger’s test, and trim and fill.

**Results:**

The quantitative synthesis pooled data across 14 RCTs. Cryotherapy significantly reduced venipuncture pain (primary effect measure: SMD [Hedges’ g] = −1.23; 95% CI: −2.03 to −0.44; *p* = 0.005; expressed on a common 0–10 pain scale for interpretability: MD = −1.97 points, 95% CI: −2.98 to −0.96) although substantial heterogeneity was observed (I^2^ = 93%). Subgroup analysis revealed that clinical condition significantly modified treatment response (*p* = 0.038), with maintenance hemodialysis patients experiencing the largest benefit (SMD = −1.87). Meta‐regression identified needle gauge as a notable variance modifier (coefficient = −0.16 per gauge unit; *p* = 0.070), suggesting enhanced relative relief with finer needles. Cryotherapy yielded no statistically significant effect on anxiety (SMD = −0.19; 95% CI: −1.49 to 1.10; *p* = 0.59; I^2^ = 75%), but was associated with a small reduction in postprocedure heart rate (MD = −2.58 bpm; 95% CI: −4.20 to −0.96; *p* = 0.015; I^2^ = 0%). According to GRADE criteria, certainty of evidence was rated low for pain and heart rate, and very low for anxiety.

**Conclusion:**

Cryotherapy may reduce venipuncture pain, with the largest possible effects in hemodialysis patients, but it does not appear to reduce procedural anxiety. High heterogeneity, the impossibility of blinding, and low certainty of evidence mean that the findings should be read with caution, and the pooled pain effect may vary in practice. Larger, standardized trials are needed to define its clinical role.

## 1. Introduction

Venipuncture is one of the most important procedures used for various purposes, which include diagnoses of illness, monitoring of chronic conditions, and administration of treatments like medications and nutrition. Despite its routine nature, it is associated with significant pain and anxiety, which can diminish the overall patient experience [1, 2]. Because it may worsen procedural discomfort and have an adverse effect on subsequent healthcare experiences, this distress is clinically relevant [3]. Needle‐related pain and fear also represent important barriers to immunization, particularly in children [4]. Furthermore, repeated exposure to this painful procedure can harm the patient’s mental health as it is directly linked to pain and anxiety.

Several solutions exist to reduce the pain of venipuncture, which includes both pharmacological interventions and nonpharmacological interventions. Pharmacological intervention includes topical anesthetics, which are local anesthetics containing creams, gels, and patches that help block pain signals from skin. Although these topical anesthetics are useful for managing pain, their application may be restricted due to additional preparation time, expense, and practical issues [5]. Therefore, there is a growing interest in nonpharmacological interventions due to their lower cost and greater feasibility in various clinical settings.

Nonpharmacological therapies for venipuncture pain management include distraction, relaxation, correct methods, and position [3]. Cryotherapy is one such nonpharmacological intervention, a cheap, simple, and accessible alternative. It involves applying cold packs, such as ice packs or other devices before the procedure to reduce pain. This technique significantly lessens the pain associated with peripheral intravenous catheterization, a needle‐related procedure closely related to venipuncture, according to recent randomized trials [6, 7].

Cryotherapy’s analgesic effects are mediated by a number of physiological processes. Vasoconstriction induced by local cooling reduces tissue blood flow and metabolic activity, which may decrease inflammatory responses and contribute to analgesic effects [8]. Additionally, cryotherapy lowers peripheral nerve conduction velocity and raises pain threshold, which delays the transmission of pain impulses and lessens pain perception [8]. Additionally, according to the gate‐control theory of pain, the stimulation of cold‐sensitive sensory fibers may prevent nociceptive signals from being transmitted at the spinal cord level, thereby contributing to analgesia [9]. When taken as a whole, these physiological effects offer a biological justification for using cryotherapy to reduce acute procedural pain during venipuncture [10]. These mechanisms might be especially important during venipuncture, as the stimulation of cutaneous nociceptors during needle insertion is the primary cause of pain.

Conventional ice packs, cold gel packs, vapocoolant sprays, and thermoelectric cooling devices are some of the methods that can be used to provide cryotherapy [10, 11]. These techniques differ in terms of cooling intensity, duration of application, tissue penetration, and ease of administration, despite having the same goal of lowering procedure discomfort by localized cooling [10]. The choice of cryotherapy modality may also be influenced by practical factors such as cost, availability, patient comfort, and feasibility in various healthcare settings [10]. When analyzing data from randomized controlled trials (RCTs), these variables should be taken into account since they may lead to variability in clinical effectiveness reported across studies [10, 11].

There is a knowledge gap and a greater need for research on cold therapy because prior studies have produced conflicting findings. Previous systematic reviews have primarily focused on psychological interventions for needle‐related pain or specific cryotherapy modalities, such as vapocoolant sprays, rather than comprehensively evaluating cryotherapy for venipuncture across different age groups [3, 11, 12]. Additionally, several RCTs evaluating different cryotherapy modalities have been published in recent years, providing new evidence that has not yet been comprehensively synthesized [6, 7]. The diversity of study populations, cryotherapy modalities, and outcome measures has further limited the generalizability of the available evidence. In order to offer a thorough assessment of the efficacy of cryotherapy in lowering pain and anxiety during venipuncture, an updated systematic review and meta‐analysis are necessary.

Effective management of venipuncture‐related pain is clinically important because poorly managed procedural pain may increase fear, anxiety, and distress, thereby negatively affecting patient cooperation and satisfaction [5, 13]. Repeated exposure to painful needle procedures may also contribute to the development of persistent needle fear and avoidance of future healthcare encounters, particularly among children and individuals requiring frequent venipuncture [13]. As a low‐cost, noninvasive, and easy‐to‐administer intervention, cryotherapy may represent a feasible strategy for procedural pain management, although further evidence is required to support its routine clinical implementation [6, 7, 11].

Therefore, the aim of this meta‐analysis is to synthesize the evidence from RCTs on the efficacy of cold application for reducing pain and anxiety during venipuncture across all age groups.

## 2. Methodology

### 2.1. Protocol and Registration

This systematic review and meta‐analysis was conducted in accordance with the Preferred Reporting Items for Systematic Reviews and Meta‐Analyses (PRISMA) guidelines [13]. The protocol was registered on PROSPERO (Registration No. CRD420251156967).

### 2.2. Literature Search Strategy

A comprehensive literature search was performed across six electronic databases: PubMed, Cochrane Library, Embase, Scopus, Web of Science, and ClinicalTrials.gov. The search strategy employed the following terms: (“ice pack” OR “cold application” OR cryotherapy) AND (venipuncture OR “blood draw” OR “IV insertion”) AND (pain OR anxiety) AND (randomized OR “randomized controlled trial” OR RCT). The search was limited to articles published between January 1, 1990, and July 1, 2026, and restricted to randomized controlled trials. The initial search yielded 502 records (PubMed: 50, Embase: 122, Cochrane: 122, Scopus: 100, Web of Science: 64, ClinicalTrials.gov: 44). Detailed search strategies for each database are given in Supporting Files. After all eligible articles were identified, they were retrieved manually (Supporting Table S1).

### 2.3. Eligibility Criteria

Eligible studies were RCTs involving human participants undergoing venipuncture or intravenous cannulation, with a cryotherapy intervention (ice packs, cold gel, vapocoolant sprays, or thermoelectric cooling bands) compared against standard care, placebo, sham device, or other comparator, and reporting pain and/or anxiety outcomes. Studies were excluded if they were nonrandomized, reviews, animal studies, or abstract‐only papers, or they involved other interventions (heat, music, distraction), addressed other procedures (surgery, vaccination, injection), or lacked pain or anxiety outcomes.

### 2.4. Study Selection and Data Extraction

Two researchers independently performed title and abstract screening using Rayyan. The same researchers also evaluated the full‐text articles against the eligibility criteria and performed data extraction. Conflicts were resolved by a third researcher. Following the systematic screening process, 303 records were screened after removing 199 duplicates, 40 full texts were assessed for eligibility, and 16 RCTs met the inclusion criteria. Of these, 14 reported sufficient data (group means with standard deviations for both arms) to be combined statistically and made up the meta‐analysis; the remaining two studies were described narratively. Baseline characteristics extracted from included articles included study ID, author, year, country, population and setting, clinical condition, cryotherapy type and application site, application time, comparator, pain scale used, and sample size per arm. Outcomes extracted included pain, anxiety, and heart rate. For studies that reported only median, interquartile range, or full range, mean and standard deviation were calculated according to the estimating formulae of Wan et al. [14].

### 2.5. Quality Assessment

Quality of included studies was assessed using the Cochrane Risk of Bias 2 (ROB2) tool for randomized trials [15]. The following domains were evaluated: randomization process (Domain 1), deviations from intended interventions (Domain 2), missing outcome data (Domain 3), measurement of the outcome (Domain 4), and selection of the reported result (Domain 5). Each domain was judged as low risk, high risk, or some concerns. Overall risk of bias was summarized per study and by domain. Two independent researchers assessed risk of bias, and conflicts were resolved by a third researcher.

### 2.6. Data Analysis

This systematic review and meta‐analysis was conducted in accordance with the recommendations of the Cochrane Collaboration [16]. For continuous outcomes, we calculated mean differences (MDs) or standardized mean differences (SMDs) with 95% confidence intervals (CIs), depending on whether studies used the same measurement scale. For pain outcomes, studies reporting on a 0–100‐mm Visual Analog Scale (VAS) were converted to a 0–10‐point scale by dividing by 10 to allow pooling with studies that used a 0–10 VAS or Numerical Rating Scale (NRS). This conversion is supported by evidence demonstrating the validity and functional equivalence of these scales for measuring pain intensity [17]. To assess statistical heterogeneity, we applied the I^2^ statistic and interpreted results according to Cochrane Handbook thresholds: 0%–40% (low), 30%–60% (moderate), 50%–90% (substantial), and 75%–100% (considerable) [18].

Given the expected clinical and methodological variability among studies, a random‐effects model was used for pooling, with the between‐study variance (τ^2^) estimated using the restricted maximum likelihood (REML) method. The Hartung–Knapp adjustment was applied for the primary analysis [19, 20]. All statistical analyses were conducted in R using the meta and metafor packages. For the primary outcome (pain), the effect measure was the SMD (Hedges′ g) to accommodate different pain scales; this was also converted to a MD on a common 0–10 scale for interpretability. The 95% prediction interval was calculated to estimate the likely range of true effects in future settings. Prespecified subgroup analyses were conducted using mixed‐effects models for categorical moderators (population, clinical condition, application site, modality type, and age group). Meta‐regression was performed for continuous moderators (application duration, mean age, needle gauge, and year of publication). Sensitivity analyses included leave‐one‐out influence analyses, restriction to local application sites, parallel‐group designs only, exclusion of high risk‐of‐bias trials, exclusion of studies with converted statistics, and use of the DerSimonian–Laird estimator instead of REML. Small‐study effects and publication bias were examined via Egger’s test and visual inspection of the funnel plot, with trim‐and‐fill adjustment applied where appropriate [21]; because more than ten trials contributed to the pain analysis, small‐study effects could be examined [19].

### 2.7. Certainty of Evidence (GRADE)

The certainty of the evidence for each outcome (pain, anxiety, and heart rate) was rated using the Grading of Recommendations, Assessment, Development and Evaluations (GRADE) framework, considering risk of bias, inconsistency, imprecision, indirectness, and publication bias [22]. Starting from high certainty because every pooled study was a randomized trial, evidence was downgraded according to established criteria.

## 3. Results

### 3.1. Study Selection

The searches returned 502 records. Removing duplicates left 303, and after screening titles and abstracts, 40 full texts were read in full against the eligibility criteria. Sixteen studies met the criteria. Of these, 14 reported enough data, that is, group means with standard deviations for both arms, to be combined statistically, and these made up the meta‐analysis. The remaining two are described narratively at the end of this section, because their design or the way they reported results ruled out pooling. Figure 1 shows the selection process (Supporting Figure S1).

**FIGURE 1:**
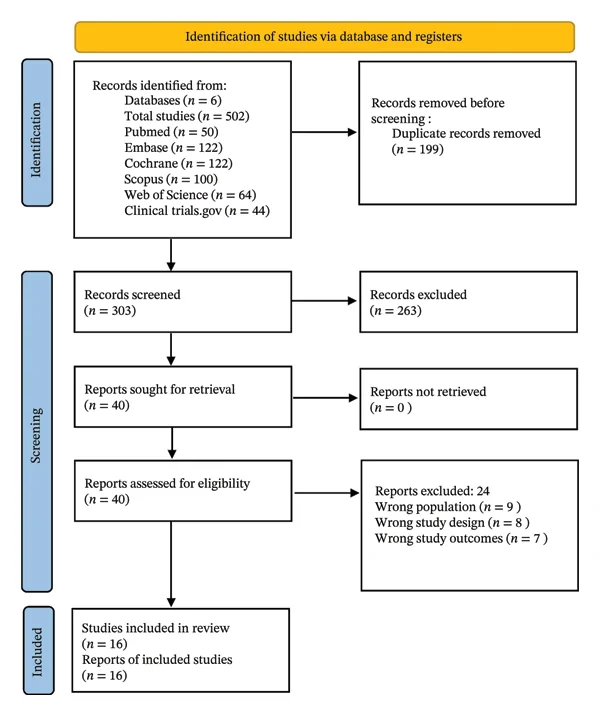
This PRISMA flow diagram systematically documents the process of study selection for a systematic review. The flow diagram clearly outlines the attrition of records at each stage, providing transparency into the study selection process.

### 3.2. Study Characteristics

Table 1 lists the individual studies. The 14 pooled trials contributed 1012 participants, divided fairly evenly between cryotherapy (507) and control (505), and were published across more than 3 decades, from 1994 to 2026. The samples were mixed. Four trials recruited children, from preschool age up to 12 years, and the rest studied adults, including healthy volunteers, patients presenting for surgery, and people on maintenance hemodialysis. The cold was applied in several ways. An ice pack or cold compress held against the puncture site was the most common approach, but four trials used a vapocoolant or cooling spray, two used a small thermoelectric cooling band, and one applied cold to the side of the neck on the premise that it would act through the vagus nerve. Comparators were usual care, no intervention, a sham device, or a placebo spray. Pain was rated on whatever scale fitted the population, most often a visual analog or numerical rating scale, with the Wong Baker Faces scale and the Face, Legs, Activity, Cry, Consolability (FLACC) scale used in the pediatric studies. Contact time ranged widely, from a spray lasting a few seconds to an ice pack left in place for about 20 minutes.

**TABLE 1:** Characteristics of the included studies (*n* = 16).

Study	Country	Population and setting	Condition	Cryotherapy type and application site	Application time	Comparator	Pain scale	Sample size (cryo vs control)
Adıgüzel 2023	Turkey	Adults, venipuncture	Non‐HD	Cold pack to lateral neck (vagal), remote	∼30 s	Sham device + routine	NRS (0–10)	56 vs 54
Aghajanloo 2016	Iran	Adults, hemodialysis	HD (AVF)	Ice to contralateral hand web (gate control), remote	3 min	Placebo (wooden cubes)	VAS (0–10)	25 vs 25
Baelen 1994	NR (French)	Children	Non‐HD	Vapocoolant (freon) spray, local	∼10 s	Placebo spray	VAS (0–100)	40 vs 40
Farion 2008	Canada	Children, emergency department	Non‐HD	Vapocoolant spray, local	∼7 s	Placebo spray (double blind)	VAS (0–100)	40 vs 40
Hartstein 2008	USA	Adults, emergency department	Non‐HD	Vapocoolant spray, local	∼3 s	No treatment (unblinded)	VAS (0–100)	47 vs 45
Hassan 2022	Malaysia	Children	Non‐HD	Ice compression, local	2 min	Routine care	VAS (0–100)	30 vs 30
Hur and Choi 2021	South Korea	Healthy adults	Non‐HD	Thermoelectric cooling band, local (wrist)	∼10 s	No‐stimulation control	NRS (0–10)	30 vs 30
Issac 2016^∗^	India	Adults, hemodialysis	HD	Ice to contralateral hand web, remote	NR	Routine care	NRS (0–10)	NR (test statistics only)
Jafarimanesh 2024	Iran	Adults, medical‐surgical	Non‐HD	Ice or cold pack, local	20 min	Control arm (multi‐arm trial)	VAS (0–10)	30 vs 30
Khosravi Pour 2023^∗^	Iran	Adults, hemodialysis	HD	Cold application to fistula (crossover), local	NR	Lidocaine; placebo	Pain (NR)	Crossover (adjusted estimate only)
Kim 2026	South Korea	Adults	Non‐HD	Thermoelectric band on upper arm, remote	10–30 s	Latex tourniquet	NRS (0–10)	31 vs 33
Korkut 2020	Turkey	Adults	Non‐HD	Ice or cold pack, local	1 min	Routine care (3‐arm trial)	NRS (0–10)	30 vs 30
Koushki 2023	Iran	Adults, hemodialysis	HD (AVF)	Cold pack, local	20 min	Routine care (3‐arm trial)	VAS (0–10)	33 vs 33
Richman 1999	USA	Healthy adult volunteers	Healthy	Ice pack, local	10 min	Paired no‐ice control	VAS (0–100)	28 vs 28 (paired)
Shafii 2020	Iran	Adults, hemodialysis	HD (AVF)	Vapocoolant spray, local	∼5–10 s	Placebo distilled‐water spray (double blind)	VAS (0–10)	40 vs 40
Sultan 2026	Pakistan	Children	Non‐HD	Ice or cold pack, local	3 min	Routine care	Wong–Baker FACES (0–10)	47 vs 47

*Note:* HD, hemodialysis; AVF, arteriovenous fistula; FLACC, Face, Legs, Activity, Cry, Consolability scale.

Abbreviations: NR, not reported; NRS, Numerical Rating Scale; VAS, Visual Analog Scale.

^∗^Study included in the review but not in the quantitative synthesis (see the text).

### 3.3. Risk of Bias Assessment

Figure 2 shows the ROB2 assessment for the 14 pooled trials. One study was judged low risk overall, eight raised some concerns, and five were high risk. The recurring weakness was blinding. A cold pack or spray is obvious to the person receiving it, so participants could rarely be masked, and because pain was self‐reported, this left the outcome measurement domain exposed; five trials were high risk on that domain and six more raised concerns. Several reports also said little about how the allocation sequence was generated or concealed, which placed them at some concern for randomization. Missing data and selective reporting were mostly not a problem. This pattern matters for interpretation, since the domain most likely to exaggerate a treatment benefit, unblinded self‐reported pain, is the one the trials handled least well (full domain‐level and summary risk‐of‐bias plots are provided in Supporting Figures S2 and S3).

**FIGURE 2:**
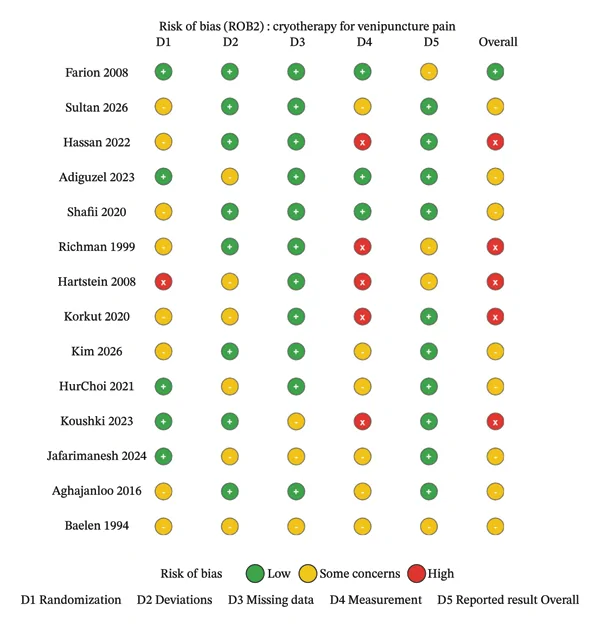
Risk of bias in the 14 pooled trials (Cochrane ROB2), by study and domain.

### 3.4. Outcomes

#### 3.4.1. Effect of Cryotherapy on Pain

Cryotherapy lowered pain during venipuncture. Pooling the 14 trials gave a SMD of −1.23 in favor of cold (95% CI −2.03 to −0.44; *p* = 0.005), from a random‐effects model with the Hartung–Knapp adjustment. The average effect is large by the usual benchmarks, but the trials disagreed a great deal: I squared reached 93%, and the between‐study variance (tau squared) was 1.75 (*Q* = 192.0 on 13 degrees of freedom, *p* < 0.001). The 95% prediction interval, which describes where the true effect in a new setting might lie, ran from −4.19 to 1.72 and so included no effect. The fair reading is that cold usually helps, and often helps a lot, but the size of the benefit is unpredictable, and in some settings, a new trial could find little or nothing. Figure 3 is the forest plot. Placed on a common 0 to 10 scale, the effect works out at a reduction of roughly 1.97 points (95% CI 0.96–2.98).

**FIGURE 3:**
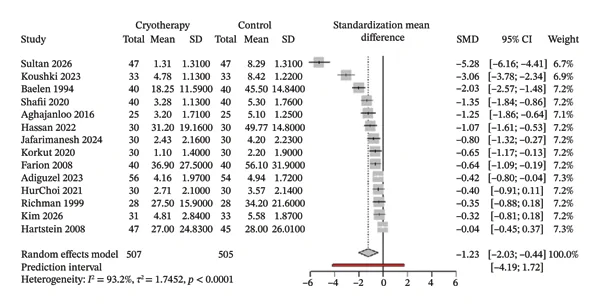
Forest plot of cryotherapy versus control for venipuncture pain (Hedges g, random effects).

##### 3.4.1.1. Subgroup Analyses

To look for the source of the disagreement, prespecified subgroups were compared (Table 2). The only split that reached significance was clinical condition (*p* = 0.038). Hemodialysis patients showed the largest average reduction, the mixed nonhemodialysis group showed a smaller but still clear one, and the single healthy volunteer trial showed the smallest. Splitting by age band, by where the cold was applied, by the type of cryotherapy, or by children versus adults did not give significant differences, although the pattern suggested that cold applied at the puncture site and delivered as ice tended to outperform cold applied elsewhere or delivered by a thermoelectric band. What stands out is that heterogeneity stayed high inside almost every subgroup, so these comparisons show where the effect was larger without really explaining why the studies differ (full subgroup results are given in Supporting Table S6 and Supporting Figures S14 to S18).

**TABLE 2:** Subgroup analyses for venipuncture pain (standardized mean difference, random effects).

Moderator	Level	*k*	SMD	Lower	Upper	I^2^_pct	p‐between
Population	Adult	9	−0.894	−1.575	−0.212	88	0.1208
	Child	4	−2.232	−5.555	1.09	96.8	0.1208
	Healthy	1	−0.348	−0.876	0.18	NA	0.1208

Condition	HD	3	−1.866	−4.365	0.634	88.7	0.0378
	Healthy	1	−0.348	−0.876	0.18	NA	0.0378
	Non‐HD	10	−1.138	−2.222	−0.054	93.9	0.0378

Site	Local	11	−1.399	−2.419	−0.378	94.4	0.1509
	Remote	3	−0.625	−1.842	0.592	68.5	0.1509

Modality class	Cold (neck/vagal)	1	−0.418	−0.796	−0.04	NA	0.0847
	Ice/cold pack	7	−1.757	−3.387	−0.126	95.2	0.0847
	Thermoelectric band	2	−0.358	−0.878	0.163	0	0.0847
	Vapocoolant spray	4	−1.002	−2.373	0.369	92.1	0.0847

Age group	< 30	5	−1.861	−4.323	0.601	96.3	0.3594
	≥ 30	8	−0.999	−1.742	−0.256	87.3	0.3594

##### 3.4.1.2. Meta‐Regression

Meta‐regression pointed the same way. Application time (*p* = 0.46), mean age (*p* = 0.30), and year of publication (*p* = 0.49) were unrelated to the size of the effect and explained essentially none of the heterogeneity. Needle gauge came closest to mattering: Across the 10 trials that recorded it, finer needles tended to accompany larger pain reductions (coefficient −0.16 per gauge unit, *p* = 0.070), and gauge on its own accounted for about a third of the variance. Since it fell short of significance and rests on a subset of trials, it is best read as a lead for future work rather than a result. As detailed in Table 3 (see also Supporting Table S5 and Supporting Figures S10–S13), a model combining duration, age, and site was not significant (*p* = 0.62).

**TABLE 3:** Meta‐regression.

Moderator	Estimate	se	*p* val	R2_pct	QMp
Duration—min	−0.0417	0.0547	0.4605	0	0.4605
Mean—age	0.0226	0.0206	0.2952	1.2	0.2952
Needle—gauge	−0.1616	0.0772	0.0697	34	0.0697
Year	−0.0275	0.0382	0.4853	0	0.4853
Multivariable	−0.0469	0.0657	0.4934	0	0.6214
Multivariable	0.025	0.0266	0.3723	0	0.6214
Multivariable	0.1925	1.1792	0.8739	0	0.6214

##### 3.4.1.3. Sensitivity Analyses

The pooled effect held up under a range of checks (Table 4). Leaving out one study at a time moved the estimate between −0.93 and −1.33, and it stayed significant every time. The most influential single trial was Sultan 2026; removing it lowered I squared to about 87%, and the influence statistics and the Baujat plot flagged the same study. Restricting the analysis to trials that applied cold at the puncture site, to parallel‐group designs, or to studies at lower risk of bias, and dropping one trial whose statistics had to be recovered from a median each left a significant benefit of much the same size. Replacing the REML estimator with DerSimonian and Laird barely changed anything. The two smallest subsets, hemodialysis and children, kept wide intervals, which is a reminder that the effect in those groups is clear in direction but loosely estimated (full outputs are provided in Supporting Table S4 and Supporting Figures S7, S8, and S9).

**TABLE 4:** Sensitivity analyses for venipuncture pain.

Analysis	*k*	SMD (95% CI)	I^2^ (%)
Primary analysis (all pooled trials)	14	−1.23 (−2.03 to −0.44)	93
Local application site only	11	−1.40 (−2.42 to −0.38)	94
Parallel designs only (drop paired trial)	13	−1.30 (−2.16 to −0.45)	94
Drop the median‐converted trial (Baelen)	13	−1.17 (−2.03 to −0.32)	93
Exclude high risk of bias trials	9	−1.36 (−2.54 to −0.18)	94
Hemodialysis population	3	−1.87 (−4.37 to 0.63)	89
Children	4	−2.23 (−5.55 to 1.09)	97
DerSimonian and Laird estimator	14	−1.22 (−2.00 to −0.43)	93
Mean difference on a common 0 to 10 scale	14	−1.97 (−2.98 to −0.96)	97

##### 3.4.1.4. Small Study Effects and Publication Bias

Because more than ten trials contributed, small‐study effects could be examined. Egger’s test was clearly significant (*t* = −5.41, *p* = 0.0002), which usually signals funnel plot asymmetry (Figure 4). The trim‐and‐fill method, however, added no studies and left the estimate unchanged. The analysis suggests that the observed asymmetry may primarily reflect a few small trials reporting unusually large effects, including Sultan 2026 and Koushki 2023, along with substantial heterogeneity, rather than the absence of negative trials. Publication bias cannot be ruled out and is listed among the limitations, but the two lines of evidence do not agree, so it was treated as a caution rather than a firm conclusion (the funnel plot, trim‐and‐fill analysis, and Egger’s test are provided in Supporting Figures S5 and S6 and Supporting Table S3).

**FIGURE 4:**
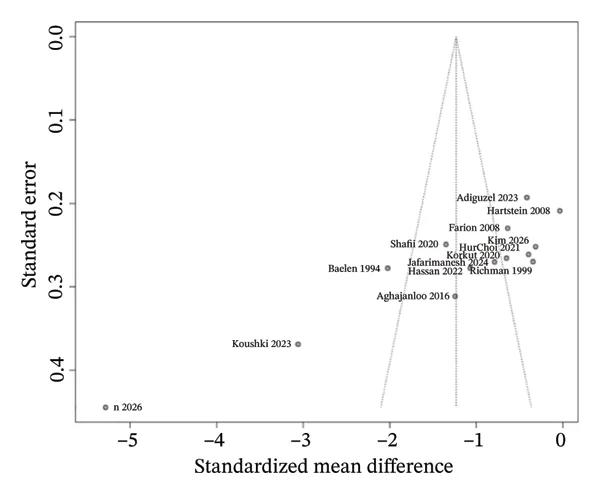
Funnel plot for venipuncture pain, with the pooled estimate and pseudo‐95% confidence limits.

#### 3.4.2. Effect of Cryotherapy on Anxiety

Only three trials (184 participants) measured anxiety. Pooled, cryotherapy made no clear difference: the standardized mean difference was −0.19 (95% CI −1.49 to 1.10; *p* = 0.59), with heterogeneity of 75%. The confidence interval extends substantially into both the benefit and harm ranges, indicating that the available evidence is insufficient to determine whether the intervention meaningfully affects anxiety during venipuncture. Therefore, the effect estimate remains too imprecise to support a definitive conclusion (Figure 5).

**FIGURE 5:**
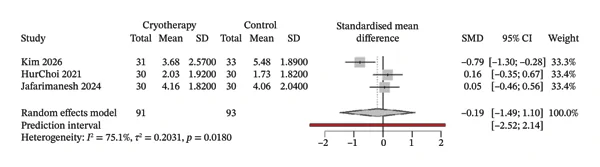
Forest plot of cryotherapy versus control for anxiety (Hedges g, random effects).

#### 3.4.3. Effect on Heart Rate

Four trials (294 participants) recorded heart rate after the procedure. Here, cold was associated with a small but consistent reduction of 2.58 beats per minute (95% CI 0.96 to 4.20 lower; *p* = 0.015), and the trials agreed closely (I squared 0%; Figure 6). Heart rate is only an indirect marker of how uncomfortable the procedure felt, so this supports the pain findings rather than adding much on its own, and it should be read as secondary.

**FIGURE 6:**
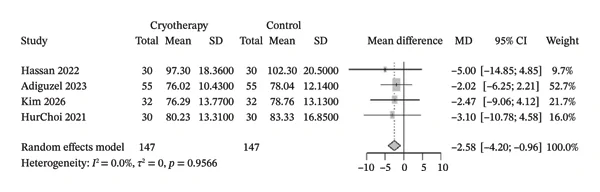
Forest plot of cryotherapy versus control for postprocedure heart rate (mean difference).

#### 3.4.4. Certainty of Evidence

Certainty was graded for each outcome, starting from high because every pooled study was a randomized trial (the domain judgements are summarized in Figure 7). Pain was rated low. It lost one level to risk of bias, mainly the unavoidable lack of blinding and the five high risk trials, and a second level to inconsistency, given the 93% I^2^ and a prediction interval that crosses no effect. The significant Egger test was noted, but because trim and fill imputed nothing, publication bias was treated as a caution rather than a further downgrade. Anxiety was rated very low, marked down for inconsistency and, more heavily, for imprecision, since it rests on three small and disagreeing trials. Heart rate was rated low, reflecting that it is a surrogate measure and that only four trials informed it (the GRADE Summary of Findings and evidence plots are provided in Supporting Table S2 and Supporting Figure S4).

**FIGURE 7:**
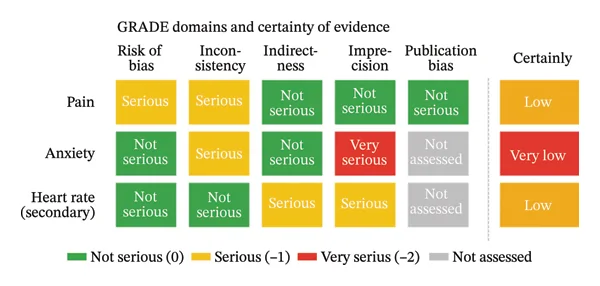
GRADE domain judgements and certainty of evidence by outcome.

##### 3.4.4.1. Studies Not Included in the Quantitative Synthesis

Two eligible studies could not be entered into the meta‐analysis. Khosravi Pour 2023 used a crossover design in which a carryover effect appeared, and it reported an adjusted regression coefficient rather than the group means and standard deviations needed for pooling. Issac and Namboothiri 2016 gave only test statistics and *p* values. Both pointed in the same direction, describing less pain with cold application, so nothing about them sits against the pooled finding; they simply could not be combined with it.

## 4. Discussion

The use of low‐temperature applications for treatment of pain and swelling (cryotherapy) is known as cold therapy [23]. While widely used in routine clinical practice to alleviate needle‐related distress, the published literature shows variable success across different clinical settings and patient demographics [4, 5]. Our meta‐analysis evaluated the effectiveness of this intervention across three distinct endpoints: pain, anxiety, and postprocedure heart rate. By examining these parameters concurrently, our analysis reveals a clear divergence between localized physical discomfort and subjective emotional stress. Cold therapy demonstrated a statistically significant reduction in venipuncture pain, whereas no significant association was found for anxiety, suggesting that, on current evidence, cold application does not appear to offer a clear advantage over standard care for procedural apprehension.

The statistically significant reduction in pain observed following cold application is corroborated by a robust pooling of 14 trials comprising 1012 participants. The overall average effect is large (SMD = −1.23; equivalent to a 1.97‐point reduction on a 0–10 scale). This analgesic magnitude aligns with broader systematic reviews on nonpharmacological physical comfort measures during invasive vascular procedures, such as those by Inal and Kelleci and Guillari et al. [24, 25]. In contrast, meta‐analyses restricted to vapocoolant sprays, a specific and briefer cryotherapy modality, have reported more modest pooled effects. Griffith et al. in their Cochrane review found that vapocoolants reduced pain at venous cannulation to only a modest and uncertain degree, with overall evidence quality rated low [10]. Similarly, Zhu et al. reported a small‐to‐moderate pooled effect for vapocoolant spray versus placebo or no treatment in intravenous cannulation [11], and Wang et al. found comparable results for vascular puncture across children and adults [8]. The larger pooled SMD observed in the present analysis likely reflects the inclusion of prolonged ice‐pack application protocols and the diversity of patient populations, particularly hemodialysis patients, who appear to experience disproportionately greater benefit, rather than a genuine superiority of cryotherapy as a class.

Furthermore, the effect size observed here compares favorably to some adult and pediatric cohorts approaches; the pooled estimates reported for topical pharmacological agents, such as lidocaine–prilocaine (EMLA) cream and liposomal lidocaine, yielded supportive results [2]. Supporting this, a Cochrane review evaluating topical anesthetics confirmed that while EMLA and amethocaine significantly reduce procedural pain across various invasive interventions, their overall evidence quality remains low to moderate, with neither agent improving first‐attempt procedural success and EMLA conferring a significant risk of transient local skin reactions (NNTH = 6) [3]. However, this central estimate should not be viewed as a guaranteed outcome in every setting. The wide 95% prediction interval (−4.19 to 1.72) indicates that treatment benefit remains highly variable in clinical practice. This is a critical nuance that earlier, smaller systematic reviews and narrative overviews frequently overlooked by asserting uniform clinical utility without accounting for between‐study heterogeneity or presenting prediction intervals [26].

It must be emphasized from the outset that all subgroup comparisons and meta‐regression analyses presented in this section are exploratory in nature, and no finding within them should be read as a causal explanation for the observed heterogeneity.

Splitting the data by broad age bands, application site, or cold modality yielded no statistically significant subgroup differences. This contrasts with traditional assumptions that age‐related nociceptive physiology fundamentally alters intervention potency. Instead, the patient’s underlying clinical condition was the sole factor that reached statistical significance (*p* = 0.038). Maintenance hemodialysis patients experienced the most pronounced average pain reduction (SMD = −1.87), followed by mixed nonhemodialysis cohorts (SMD = −1.14), while the single healthy volunteer trial reported the smallest benefit (SMD = −0.35). This trend suggests the hypothesis that individuals undergoing frequent, chronic vascular access, such as end‐stage kidney disease patients, may have altered baseline pain thresholds or heightened responsiveness to counter‐irritant sensory stimulation, a phenomenon also observed in primary trials evaluating arteriovenous fistula cannulation [27, 28]. Nevertheless, marked heterogeneity (I^2^ > 88%) persisted within nearly all subgroups, demonstrating that patient population alone does not fully account for interstudy variance.

In a similar vein, our meta‐regression analyses offer preliminary leads rather than conclusive findings. While publication year, patient age, and application duration explained essentially none of the variance, needle gauge emerged as a potential moderator. Meta‐regression revealed that finer needle gauges (higher gauge numbers) tended with larger pain reductions (coefficient = −0.16 per gauge unit). Although this trend did not reach standard thresholds for statistical significance (*p* = 0.070) and relies on a subset of 10 studies, it raises a preliminary hypothesis that cryotherapy may offer relatively greater relief when baseline tissue trauma is minimized by smaller‐diameter needles. This variable deserves prospective control in future primary trials, as needle dimensions are rarely accounted for or reported in existing procedural synthesis literature [29].

It is important to note at the outset that the anxiety analysis rests on an extremely limited evidence base: Only three studies contributed a total of 184 participants, which substantially limits statistical power and the generalizability of any inference. The nonsignificant pooled effect (SMD = −0.19; 95% CI: −1.49 to 1.10) may reflect a true absence of effect, insufficient power, or both, and no definitive conclusions can be drawn from these data alone. Therefore, divergence between pain and anxiety warrants detailed examination, as pain relief did not translate into reduced psychological distress (SMD = −0.19; 95% CI: −1.49 to 1.10; *p* = 0.59, I^2^ = 75%). Several methodological and neurobiological factors likely account for this disconnect:

i. Temporal Sequence of Assessment: Across the majority of included trials, anxiety was evaluated prior to or during needle insertion (anticipatory distress), whereas pain was recorded after needle entry. Cryotherapy operates primarily through rapid localized blockade of A‐delta and C nerve fibers (gate‐control mechanism), a peripheral action that cannot retroactively reduce central apprehension established before the physical stimulus occurs [7].
ii. Measurement Instruments and Variance: The three contributing studies used different assessment tools (such as the Visual Analog Scale for Anxiety versus the State‐Trait Anxiety Inventory), contributing to substantial statistical and clinical variability (I^2^ = 75%), a discrepancy previously underscored by Bringuier et al. in their psychometric evaluation of pediatric anxiety instruments [30]. Brief single‐item self‐reports may also lack the sensitivity required to separate the physical sensation of intense thermal cold from genuine procedural fear.
iii. Neurobiological Mechanisms: Although earlier primary studies hypothesized that local cooling might trigger parasympathetic tone or serve as a cognitive distraction [12, 31], our findings align with clinical models of procedural stress (Gahlawat et al.) showing that anticipatory apprehension is governed by central amygdala processing, which is largely unaffected by thermal modulation at the skin surface [32].

In contrast to self‐reported anxiety, postprocedure heart rate showed a small but statistically significant decrease (MD = −2.58 bpm; 95% CI: −4.20 to −0.96; *p* = 0.015) with no statistical heterogeneity (I^2^ = 0%). Rather than reflecting emotional calming, this attenuation likely represents a modest autonomic reflex (such as localized cold‐induced parasympathetic activation) consistent with physiological literature on thermal applications [31, 33].

Sensitivity analyses confirmed that the overall pooled pain effect is highly resilient across leave‐one‐out iterations, design restrictions, and alternative estimation models (DerSimonian–Laird). However, in light of the persistent unexplained heterogeneity, evidence of small‐study effects on Egger’s test (*p* = 0.002), and the low‐to‐very‐low GRADE certainty ratings across outcomes, these conclusions offer indicative guidance rather than definitive clinical rules. Future primary trials should standardize cold application protocols, clearly separate preprocedural anxiety from postprocedural pain, and routinely report needle specifications to better define the clinical utility of cryotherapy [19].

### 4.1. Strengths of the Meta‐Analysis

By limiting our inclusion criteria strictly to RCTs and adhering rigorously to PRISMA guidelines, we ensured high methodological standards. Pooling data from 14 trials (1012 participants) for pain outcomes substantially increased the precision of the overall effect size estimation compared to individual, isolated studies. Furthermore, conducting comprehensive sensitivity analyses, which confirmed that the pooled effect remained significant even when isolating parallel designs or accounting for highly influential trials like Sultan 2026, demonstrates the mathematical resilience of our primary direction of effect.

### 4.2. Limitations

Our findings should be interpreted in accordance with several key methodological and clinical limitations:

A. Inability to Blind Participants: Due to the physical nature of cold application, participant and operator blinding was unfeasible across trials, introducing potential performance and detection bias, particularly for subjective endpoints like pain and anxiety.
B. Heterogeneity of Interventions and Comparators: Cryotherapy protocols varied significantly in application duration, device type, and temperature control. Similarly, comparator arms ranged from passive controls (no treatment or standard care) to active sham interventions, complicating head‐to‐head comparisons.
C. Variability in Outcome Measurement: The inclusion of diverse, self‐reported pain intensity scales (VAS), NRS, and pediatric behavioral scales) across trials required score standardization, which may have introduced measurement variability.
D. Constrained Anxiety Evidence: Analysis of anxiety was limited to 3 studies with a small sample size of 184 participants, substantially constraining statistical power and generalizability across broader clinical populations.
E. Exploratory Meta‐Regression and Subgroup Analyses: Multivariable meta‐regression (*p* = 0.62) and subgroup comparisons were exploratory. These analyses were constrained by incomplete procedural reporting in the primary literature such as precise needle gauge data being reported in only 10 trials, leaving residual variance unexplained.
F. Small‐Study Effects and Publication Bias: Egger’s test was statistically significant (*p* = 0.0002), suggesting possible funnel plot asymmetry; however, the trim‐and‐fill method imputed no additional studies, leaving the pooled estimate unchanged. These discordant findings preclude a definitive conclusion about publication bias, and the possibility that small studies with disproportionately large effects have inflated the pooled estimate cannot be excluded.

### 4.3. Clinical and Research Implications

The clinical implication of the present meta‐analysis is that cold therapy provides better pain regulation for patients undergoing venipuncture, although anxiety resolution remains a question. The advantages of such therapy in terms of effectiveness over standard therapy groups points to the possible usage of such intervention in future Trial‐to‐trial differences in benefit extent, however, highlight that pain reduction has been a common observation among many studies. To confirm these findings, there is a pressing need to have larger, better‐powered trials with harmonized study endpoints to define the issue of how and whether it is best to use cold therapy as an adjunct to venipuncture.

## 5. Conclusion

The available evidence suggests that local cold application may provide modest pain reduction during venipuncture; however, it does not appear to confer a clear benefit for procedural anxiety. Given the substantial clinical heterogeneity, nonblinded designs, and reliance on small trial cohorts, these results should be interpreted with caution. Cold therapy may not be sufficient as a standalone monotherapy, but it may represent a useful adjunctive component within multimodal analgesia protocols. Larger, randomized controlled trials with standardized application protocols are needed to establish its precise therapeutic role.

## Funding

No funding was received for this manuscript.

## Conflicts of Interest

The authors declare no conflicts of interest.

## Supporting Information

Additional supporting information can be found online in the Supporting Information section.

## Supporting information


**Supporting Information** The supporting information provides the full methodological and analytical detail supporting this review. Supporting Table S1 presents the complete electronic search strategies for all databases and registries, and Supporting Figure S1 shows the PRISMA 2020 study selection flow diagram. Supporting Figures S2 and S3 give the domain‐level and summary risk‐of‐bias (ROB2) plots. Supporting Table S2 and Supporting Figure S4 present the GRADE Summary of Findings and evidence plots. Publication bias and small‐study effects are documented in Supporting Figures S5 and S6 and Supporting Table S3 (funnel plot, trim‐and‐fill, and Egger’s test). Sensitivity analyses are provided in Supporting Table S4 and Supporting Figures S7 to S9 (leave‐one‐out, Baujat, and influence diagnostics). Meta‐regression outputs appear in Supporting Table S5 and Supporting Figures S10 to S13, and the subgroup analyses in Supporting Table S6 and Supporting Figures S14 to S18.

## Data Availability

The data that support the findings of this study are available from the corresponding author upon reasonable request.
