# Supplementary material for: The Efficacy of Cryotherapy in Reducing Pain and Anxiety During Venipuncture: A Systematic Review and Meta‐Analysis of Randomized Controlled Trials
Source: Pain Res Manag. 2026 Nov 9;2026:9474988. doi: 10.1155/prm/9474988 (PMC13748115; doi:10.1155/prm/9474988)
Supplement: Supplementary file 1 [file PRM-2026-9474988-s001.docx]

**Supplementary file**

**For**

**The Efficacy of Cryotherapy in Reducing Pain and Anxiety During Venipuncture: A Systematic Review and Meta-Analysis of Randomized Controlled Trials.**

## **Supplementary Material**

## **Contents:**

### **Supplementary Table S1.**

**Complete electronic search strategies used for all databases and trial registries.**

### **Supplementary Figure S1.**

**PRISMA 2020 flow diagram illustrating the study selection process.**

### **Supplementary Figure S2.**

**Risk of Bias (ROB 2) traffic-light plot showing domain-level risk of bias judgments for each included randomized controlled trials.**

### **Supplementary Figure S3.**

**Risk of Bias (ROB 2) summary plot showing the overall distribution of risk of bias judgments across all included studies.**

### **Supplementary Table S2.**

**Summary of Findings (SoF) table generated using the GRADE approach.**

### **Supplementary Figure S4**

GRADE assessment evidence plots

## **Publication Bias**

### **Supplementary Figure S5.**

**Funnel plot assessing potential publication bias for the primary outcome (pain).**

### **Supplementary Figure S6.**

**Trim-and-fill analysis evaluating the potential impact of unpublished studies on the pooled effect estimate.**

### **Supplementary Table S3.**

**Results of Egger's regression test for publication bias and small-study effects.**

## **Sensitivity Analysis**

## Supplementary Table S4

Leave-one-out sensitivity analysis

### **Supplementary Figure S7.**

**Leave-one-out sensitivity analysis evaluating the influence of individual studies on the pooled effect estimate.**

### **Supplementary Figure S8.**

**Baujat plot identifying studies contributing most to heterogeneity and overall influence on the pooled analysis.**

### **Supplementary Figure S9.**

**Influence diagnostics plot assessing influential studies in the meta-analysis.**

## **Meta-regression Analysis**

## Supplementary Table S5.

Results of univariable meta-regression analyses exploring potential sources of heterogeneity.

### **Supplementary Figure S10.**

**Bubble plot of univariable meta-regression according to cryotherapy duration.**

### **Supplementary Figure S11.**

**Bubble plot of univariable meta-regression according to participants' mean age.**

### **Supplementary Figure S12.**

**Bubble plot of univariable meta-regression according to needle gauge.**

### **Supplementary Figure S13.**

**Bubble plot of univariable meta-regression according to publication year.**

## **Subgroup Analyses**

### **Supplementary Table S6.**

**Summary of subgroup analyses for the primary outcome.**

### **Supplementary Figure S14.**

**Forest plot of subgroup analysis according to study population.**

### **Supplementary Figure S15.**

**Forest plot of subgroup analysis according to clinical condition.**

### **Supplementary Figure S16.**

**Forest plot of subgroup analysis according to cryotherapy application site.**

### **Supplementary Figure S17.**

**Forest plot of subgroup analysis according to cryotherapy modality.**

### **Supplementary Figure S18.**

**Forest plot of subgroup analysis according to age group.**

**Supplementary Table S1.**

**Complete electronic search strategies used for all databases and trial registries**.

| Database | Records Identified (n) | Date Searched | Search Strategy |
| --- | --- | --- | --- |
| PubMed | 50 | 29 June 2026 | ("Cryotherapy"[Mesh] OR cryotherap*[tiab] OR cryoanalgesi*[tiab] OR "cold therap*"[tiab] OR "cold application*"[tiab] OR "cold compress*"[tiab] OR "cold pack*"[tiab] OR "ice pack*"[tiab] OR "ice therap*"[tiab] OR "ice application*"[tiab] OR cooling[tiab] OR "cooling device*"[tiab]) AND ("Venipuncture"[Mesh] OR venipuncture*[tiab] OR phlebotom*[tiab] OR "blood draw*"[tiab] OR "blood collection"[tiab] OR "blood sampling"[tiab] OR "venous puncture"[tiab] OR cannulation[tiab] OR catheterization[tiab] OR "intravenous catheter*"[tiab] OR "IV insertion"[tiab] OR "vascular access"[tiab]) AND ("Pain"[Mesh] OR pain[tiab] OR painful[tiab] OR "pain intensity"[tiab] OR "pain score*"[tiab] OR VAS[tiab] OR "visual analog scale"[tiab] OR "visual analogue scale"[tiab] OR NRS[tiab] OR "numeric rating scale"[tiab] OR "Anxiety"[Mesh] OR anxi*[tiab] OR fear[tiab] OR distress[tiab]) AND (randomized controlled trial[pt] OR controlled clinical trial[pt] OR randomized[tiab] OR randomised[tiab] OR randomly[tiab] OR placebo[tiab] OR trial[ti]) NOT (animals[mh] NOT humans[mh]) |
| Embase | 122 | 29 June 2026 | ('cryotherapy'/exp OR cryotherap*:ti,ab,kw OR cryoanalgesi*:ti,ab,kw OR 'cold therapy':ti,ab,kw OR 'cold application':ti,ab,kw OR 'cold compress':ti,ab,kw OR 'ice pack':ti,ab,kw OR 'ice therapy':ti,ab,kw OR cooling:ti,ab,kw) AND ('venipuncture'/exp OR venipuncture*:ti,ab,kw OR phlebotom*:ti,ab,kw OR 'blood sampling':ti,ab,kw OR 'blood draw':ti,ab,kw OR cannulation:ti,ab,kw OR catheterization:ti,ab,kw OR 'vascular access':ti,ab,kw OR 'intravenous catheter':ti,ab,kw) AND ('pain'/exp OR pain*:ti,ab,kw OR 'pain intensity':ti,ab,kw OR anxiety/exp OR anxi*:ti,ab,kw OR distress:ti,ab,kw OR fear:ti,ab,kw) AND ('randomized controlled trial'/exp OR random*:ti,ab,kw OR placebo*:ti,ab,kw) |
| Scopus | 100 | 29 June 2026 | TITLE-ABS-KEY (Cryotherap* OR cryoanalgesi* OR "cold therapy" OR "cold application" OR "cold compress" OR "ice pack" OR "ice therapy" OR cooling) AND TITLE-ABS-KEY (Venipuncture* OR phlebotom* OR "blood draw" OR "blood sampling" OR "blood collection" OR cannulation OR catheterization OR "vascular access" OR "intravenous catheter") AND TITLE-ABS-KEY (Pain OR painful OR "pain intensity" OR anxiety OR distress OR fear) AND TITLE-ABS-KEY (Random* OR placebo OR trial) |
| Web of Science | 64 | 29 June 2026 | TS=(Cryotherap* OR cryoanalgesi* OR "cold therapy" OR "cold application" OR "cold compress" OR "ice pack" OR cooling) AND TS=(Venipuncture* OR phlebotom* OR "blood draw" OR "blood sampling" OR cannulation OR catheterization OR "vascular access") AND TS=(Pain OR anxiety OR distress OR fear) AND TS=(Random* OR placebo OR trial) |
| Cochrane CENTRAL | 122 | 29 June 2026 | (Cryotherap* OR cryoanalgesi* OR "cold therapy" OR "cold application" OR "cold compress" OR "ice pack") AND (Venipuncture* OR phlebotom* OR "blood draw" OR "blood sampling" OR cannulation OR catheterization OR "vascular access") AND (Pain OR anxiety OR fear OR distress) |
| ClinicalTrials.gov | 44 | 29 June 2026 | (Venipuncture OR Blood Sampling OR Phlebotomy) AND (Cryotherapy OR Ice Pack OR Cold Application OR Cold Therapy) |

**Supplementary Figure S1.**

**PRISMA 2020 flow diagram illustrating the study selection process.**

**Identification of studies via databases and registers**

Records identified from*:

Databases (n =6 )

Total studies (n= 502)

Pubmed (n= 50)

Embase (n=122)

Cochrane (n=122)

Scopus (n= 100)

Web of science(n=64)

Clinical trials.gov (n= 44)

Records removed *before screening*:

Duplicate records removed (n = 199 )

**Identification**

Records screened

(n =303 )

Records excluded**

(n = 263)

Reports sought for retrieval

(n = 40)

Reports not retrieved

(n = 0 )

**Screening**

Reports assessed for eligibility

(n = 40 )

Reports excluded: 24

Wrong population (n = 9 )

Wrong study design (n =8 )

Wrong study outcomes (n = 7 )

Studies included in review

(n = 16 )

Reports of included studies

(n = 16 )

**Included**

**Supplementary Figure S2.**

**Risk of Bias (ROB 2) traffic-light plot showing domain-level risk of bias judgments for each included randomized controlled trial.**

**Supplementary Figure S3.**

**Risk of Bias (ROB 2) summary plot showing the proportion of studies judged as low risk, some concerns, or high risk across each bias domain.**

**Supplementary Table S2**

**Summary of Findings table generated using the GRADE approach.**

# **GRADE Summary of Findings:**

| **Outcome** | **Participants (studies)** | **Effect estimate (95% CI)** | **Absolute effect / interpretation** | **Certainty (GRADE)** | **Reasons for rating** |
| --- | --- | --- | --- | --- | --- |
| **Pain** | 1012 (14 RCTs)  507 vs 505 | SMD −1.23 (−2.03 to −0.44); p = 0.005 | About 1.97 points lower on a 0 to 10 scale (0.96 to 2.98 lower) | **Low** | Risk of bias a; inconsistency b |
| **Anxiety** | 184 (3 RCTs)  91 vs 93 | SMD −0.19 (−1.49 to 1.10); p = 0.59 | Trivial, no clear difference (\|SMD\| < 0.2) | **Very low** | Inconsistency b; serious imprecision c |
| **Heart rate (secondary)** | 294 (4 RCTs)  147 vs 147 | MD −2.58 bpm (−4.20 to −0.96); p = 0.015 | 2.58 beats per minute lower after the procedure | **Low** | Indirectness d; imprecision e |

**Supplementary Figure S4**

**GRADE assessment evidence plots**

**Supplementary Figure S5**

**Funnel plot assessing potential publication bias for the primary outcome.**

**.**

**Supplementary Figure S6**

**Trim-and-fill analysis evaluating the potential impact of unpublished studies on the pooled estimate.**

**Supplementary Table S3.**

**Results of Egger’s regression test for small-study effects and publication bias**

| Pain Egger’s linear regression analysis | | | | |
| --- | --- | --- | --- | --- |
| Test | statistic | df | p_value | bias_estimate |
| Egger linreg | -5.409 | 12 | 0.000158 | -16.578 |

**Supplementary Table S4**

**Leave-one-out sensitivity analysis**

| Leave-One-Out sensitivity analysis for pain | | | | |
| --- | --- | --- | --- | --- |
| omitted | SMD | lower | upper | I2_pct |
| Omitting Farion2008 | -1.283 | -2.144 | -0.421 | 93.7 |
| Omitting Sultan2026 | -0.928 | -1.418 | -0.438 | 86.9 |
| Omitting Hassan2022 | -1.249 | -2.117 | -0.382 | 93.7 |
| Omitting Adiguzel2023 | -1.3 | -2.155 | -0.445 | 93.5 |
| Omitting Shafii2020 | -1.227 | -2.095 | -0.359 | 93.6 |
| Omitting Richman1999 | -1.304 | -2.157 | -0.451 | 93.6 |
| Omitting Hartstein2008 | -1.329 | -2.168 | -0.489 | 93.1 |
| Omitting Korkut2020 | -1.281 | -2.143 | -0.42 | 93.7 |
| Omitting Kim2026 | -1.307 | -2.158 | -0.455 | 93.6 |
| Omitting HurChoi2021 | -1.3 | -2.155 | -0.446 | 93.6 |
| Omitting Koushki2023 | -1.096 | -1.897 | -0.296 | 92.3 |
| Omitting Jafarimanesh2024 | -1.27 | -2.135 | -0.406 | 93.7 |
| Omitting Aghajanloo2016 | -1.235 | -2.103 | -0.368 | 93.7 |
| Omitting Baelen1994 | -1.175 | -2.03 | -0.319 | 93.1 |

**Supplementary Figure S7.**

**Leave-one-out sensitivity analysis evaluating the influence of individual studies on the pooled effect estimate.**

**Supplementary Figure S8.**

**Baujat plot identifying studies contributing most to heterogeneity and overall influence on the meta-analysis.**

**Supplementary Figure S9**

**Influence diagnostics plot assessing the influence of individual studies on the pooled effect estimate**

**Supplementary Table S5.**

**Results of univariable meta-regression analyses for potential sources of heterogeneity.**

| Pain Meta-regression analysis | | | | | | |
| --- | --- | --- | --- | --- | --- | --- |
| moderator | term | estimate | se | pval | R2_pct | QMp |
| duration_min | intrcpt | -1.0581 | 0.4418 | 0.0338 | 0 | 0.4605 |
| duration_min | duration_min | -0.0417 | 0.0547 | 0.4605 | 0 | 0.4605 |
| mean_age | intrcpt | -2.0931 | 0.7929 | 0.023 | 1.2 | 0.2952 |
| mean_age | mean_age | 0.0226 | 0.0206 | 0.2952 | 1.2 | 0.2952 |
| needle_gauge | intrcpt | 2.6378 | 1.5792 | 0.1334 | 34 | 0.0697 |
| needle_gauge | needle_gauge | -0.1616 | 0.0772 | 0.0697 | 34 | 0.0697 |
| year | intrcpt | 54.2757 | 77.099 | 0.4949 | 0 | 0.4853 |
| year | year | -0.0275 | 0.0382 | 0.4853 | 0 | 0.4853 |
| multivariable | intrcpt | -2.0047 | 0.8515 | 0.043 | 0 | 0.6214 |
| multivariable | duration_min | -0.0469 | 0.0657 | 0.4934 | 0 | 0.6214 |
| multivariable | mean_age | 0.025 | 0.0266 | 0.3723 | 0 | 0.6214 |
| multivariable | siteremote | 0.1925 | 1.1792 | 0.8739 | 0 | 0.6214 |

**Meta-regression outputs: Supplementary Figure S10.**

**Bubble plot of univariable meta-regression according to cryotherapy duration.**

**Supplementary Figure S11.**

**Bubble plot of univariable meta-regression according to participants’ mean age..**

**Supplementary Figure S12.**

**Bubble plot of univariable meta-regression according to needle gauge.**

**Supplementary Figure S13.**

**Bubble plot of univariable meta-regression according to publication year**

**Supplementary Table S6.**

**Summary of subgroup analyses for the primary outcome**

| Pain Sub-group analysis | | | | | | | |
| --- | --- | --- | --- | --- | --- | --- | --- |
| moderator | level | k | SMD | lower | upper | I2_pct | p_between |
| population | adult | 9 | -0.894 | -1.575 | -0.212 | 88 | 0.1208 |
| population | child | 4 | -2.232 | -5.555 | 1.09 | 96.8 | 0.1208 |
| population | healthy | 1 | -0.348 | -0.876 | 0.18 | NA | 0.1208 |
| condition | HD | 3 | -1.866 | -4.365 | 0.634 | 88.7 | 0.0378 |
| condition | healthy | 1 | -0.348 | -0.876 | 0.18 | NA | 0.0378 |
| condition | nonHD | 10 | -1.138 | -2.222 | -0.054 | 93.9 | 0.0378 |
| site | local | 11 | -1.399 | -2.419 | -0.378 | 94.4 | 0.1509 |
| site | remote | 3 | -0.625 | -1.842 | 0.592 | 68.5 | 0.1509 |
| modality_class | Cold (neck/vagal) | 1 | -0.418 | -0.796 | -0.04 | NA | 0.0847 |
| modality_class | Ice/cold pack | 7 | -1.757 | -3.387 | -0.126 | 95.2 | 0.0847 |
| modality_class | Thermoelectric band | 2 | -0.358 | -0.878 | 0.163 | 0 | 0.0847 |
| modality_class | Vapocoolant spray | 4 | -1.002 | -2.373 | 0.369 | 92.1 | 0.0847 |
| age_group | <30 | 5 | -1.861 | -4.323 | 0.601 | 96.3 | 0.3594 |
| age_group | >=30 | 8 | -0.999 | -1.742 | -0.256 | 87.3 | 0.3594 |

**Supplementary Figure S14.**

**Forest plot of subgroup analysis according to study population..**

**Supplementary Figure S15.**

**Forest plot of subgroup analysis according to clinical condition.**

**Supplementary Figure S16.**

**Forest plot of subgroup analysis according to cryotherapy application site.**

**Supplementary Figure S17.**

**Forest plot of subgroup analysis according to cryotherapy modality.**

**Supplementary Figure S18.**

**Forest plot of subgroup analysis according to age group**
